# Supplementary material for: Identification of host proteins differentially associated with HIV-1 RNA splice variants
Source: eLife. 2021 Feb 25;10:e62470. doi: 10.7554/eLife.62470 (PMC7906601; doi:10.7554/eLife.62470)
Supplement: Figure 1—source data 1. — Related to Figure 1 and Figure 1—figure supplement 1. [file elife-62470-fig1-data1.docx]

**Figure 1-source data 1:** qPCR data for A. capture specificity B. enrichment and C. capture efficiency calculations. Related to Figure 1 and Figure S1.

1. Capture Specificity

| Capture Sample Type | Intron-1 amol | Intron-2 amol | 3’-exon amol | Intron-1 Normalized | Intron-2 Normalized | 3’-exon Normalized |
| --- | --- | --- | --- | --- | --- | --- |
| US 1-1 | 1331 | 952 | 1455 | 1.00 | 0.71 | 1.09 |
| US 1-2 | 1540 | 1088 | 1826 | 1.00 | 0.71 | 1.19 |
| US 2-1 | 1550 | 1046 | 1702 | 1.00 | 0.68 | 1.10 |
| US 2-2 | 1453 | 999 | 1493 | 1.00 | 0.69 | 1.03 |
| US 3-1 | 1277 | 846 | 1205 | 1.00 | 0.66 | 0.94 |
| US 3-2 | 1306 | 888 | 1488 | 1.00 | 0.68 | 1.14 |
| PS 1-1 | 204 | 4384 | 2587 | 0.05 | 1.00 | 0.59 |
| PS 1-2 | 199 | 3859 | 2521 | 0.05 | 1.00 | 0.65 |
| PS 2-1 | 224 | 4327 | 2313 | 0.05 | 1.00 | 0.53 |
| PS 2-2 | 222 | 4427 | 2269 | 0.05 | 1.00 | 0.51 |
| PS 3-1 | 238 | 4680 | 2328 | 0.05 | 1.00 | 0.50 |
| PS 3-2 | 219 | 4298 | 2662 | 0.05 | 1.00 | 0.62 |
| CS 1-1 | 9 | 49 | 693 | 0.01 | 0.07 | 1.00 |
| CS 1-2 | 8 | 48 | 727 | 0.01 | 0.07 | 1.00 |
| CS 2-1 | 11 | 57 | 816 | 0.01 | 0.07 | 1.00 |
| CS 2-2 | 10 | 54 | 768 | 0.01 | 0.07 | 1.00 |
| CS 3-1 | 9 | 50 | 636 | 0.01 | 0.08 | 1.00 |
| CS 3-2 | 8 | 47 | 499 | 0.02 | 0.09 | 1.00 |

1. Enrichment

| Capture Sample Type | Intron-1 amol | Intron-2 amol | 3’-exon amol | GAPDH amol | Ratio US: GAPDH | Ratio PS: GAPDH | Ratio CS: GAPDH |
| --- | --- | --- | --- | --- | --- | --- | --- |
| US1 | 332.43 |  |  | 8.73 | 38.09 |  |  |
| US2 | 404.75 |  |  | 10.46 | 38.69 |  |  |
| US3 | 295.22 |  |  | 9.75 | 30.27 |  |  |
| PS1 |  | 1214.65 |  | 11.65 |  | 104.22 |  |
| PS2 |  | 1790.38 |  | 15.61 |  | 114.68 |  |
| PS3 |  | 2136.28 |  | 12.54 |  | 170.38 |  |
| CS1 |  |  | 468.75 | 3.81 |  |  | 123.11 |
| CS2 |  |  | 621.94 | 5.33 |  |  | 116.63 |
| CS3 |  |  | 475.10 | 5.22 |  |  | 91.10 |
| Lys1 Dil10 | 0.03 | 0.24 | 0.05 | 0.23 | 0.15 | 1.02 | 0.20 |
| Lys2 Dil10 | 0.03 | 0.19 | 0.04 | 0.22 | 0.16 | 0.90 | 0.20 |
| Lys3 DIl10 | 0.04 | 0.18 | 0.06 | 0.25 | 0.15 | 0.73 | 0.23 |

1. Capture Efficiency

| Capture Sample Type | Intron-1 amol | Intron-2 amol | 3’-exon amol | Intron-2 Corrected ((Intron-2) – (Intron-1)) | 3’-exon Corrected ((3’-exon) – (Intron-2)) |
| --- | --- | --- | --- | --- | --- |
| Post 1-1 | 662 | 1153 | 6526 | 491 | 5373 |
| Post 1-2 | 737 | 1293 | 7252 | 556 | 5959 |
| Post 2-1 | 847 | 1255 | 7657 | 409 | 6401 |
| Post 2-2 | 820 | 1251 | 7584 | 432 | 6332 |
| Post 3-1 | 849 | 1440 | 9364 | 591 | 7924 |
| Post 3-2 | 1011 | 1691 | 8926 | 679 | 7235 |
| Pre 1-1 | 3708 | 7864 | 36073 | 4156 | 28209 |
| Pre 1-2 | 3817 | 7366 | 36889 | 3549 | 29523 |
| Pre 2-1 | 3817 | 7512 | 37844 | 3695 | 30332 |
| Pre 2-2 | 4634 | 8703 | 38824 | 4069 | 30121 |
| Pre 3-1 | 3732 | 8341 | 40601 | 4609 | 32260 |
| Pre 3-2 | 3880 | 8963 | 39957 | 5084 | 30994 |
