# Supplementary material for: Identification of host proteins differentially associated with HIV-1 RNA splice variants
Source: eLife. 2021 Feb 25;10:e62470. doi: 10.7554/eLife.62470 (PMC7906601; doi:10.7554/eLife.62470)
Supplement: Supplementary file 1. — Related to Figure 1 and Figure 1—figure supplement 1. [file elife-62470-supp1.docx]

**Supplementary File 1:** HIV-1 splice variant capture oligonucleotide and qPCR assay sequences and genomic locations. (Related to Figure 1 and Figure Supplement 1)

|  | US RNA | PS RNA | CS RNA |
| --- | --- | --- | --- |
| CO Name | Intron-1 CO | Intron-2 CO | 3’-Exon CO |
| CO Sequence (5’-3’) – Biotinylated | CAATTGACTTACTATGCTTTCTGTGGCTATTTTTTGTA | CAATTGACTGTACAATTAATTTCTACAGATGTGTTCAG | TCGTATCTCTTTTCTTTTAAAAAGTGGCTAAGATCTAC |
| RO Sequence (5’-3’) | TACAAAAAATAGCCACAGAAAGCATAGTAAGTCAATTG | CTGAACACATCTGTAGAAATTAATTGTACAGTCAATTG | GTAGATCTTAGCCACTTTTTAAAAGAAAAGAGATACGA |
| Complementary Location in Genome (NL4-3) | 3210-3239 | 6622-6651 | 8585-8614 |
| Amount of CO Used in Capture Experiments (pmol per 5x10^7^ cells) | 800 | 3200 | 400 |
| Amount of Beads Used per Capture (mL) | 2.4 | 4.8 | 1.2 |
| Amount of RO Used in Capture Experiments (nmol per 5x10^7^ cells) | 40 | 160 | 20 |
| qPCR Forward Primer | GAG TTT GTC AAT ACC CCT CCC | CCT CCC ATC AGT GGA CAA ATT A | AGA GGC CAA TAA AGG AGA GAA C |
| qPCR Reverse Primer | CCT CTG TCA GTT ACA TAT CCT GC | CTG AAG ATC TCG GAC CCA TTG | GCT GTC AAA CCT CCA CTC TAA |
| qPCR Probe | /56-FAM/TT TCC CTA T/Zen/T GGC TGC CCC ATC T/3IABkFQ/ | /56-FAM/AC CAC CAT C/Zen/T CTT GTT AAT AGC AGC CC/3IABkFQ/ | /56-FAM/TG TGA GCC T/Zen/G CAT GGA ATG GAT GA/3IABkFQ/ |
| qPCR Amplification Region | 3338-3480 | 7069-7166 | 8794-8893 |

Red lettering in CO and RO sequences are for toe-hold release.
