# Supplementary material for: Identification of host proteins differentially associated with HIV-1 RNA splice variants
Source: eLife. 2021 Feb 25;10:e62470. doi: 10.7554/eLife.62470 (PMC7906601; doi:10.7554/eLife.62470)
Supplement: Supplementary file 3. — Related to Figure 3. [file elife-62470-supp3.docx]

**Supplementary File 3:** siRNA sequences used for gene specific knock down screen. Related to Figure 3.

| Gene | GeneAccession | GINumber | Sequence |
| --- | --- | --- | --- |
| AARS2 | NM_020745 | 145199235 | GGGACGCACCUGUUACGUA |
| ACACA | NM_198834 | 38679959 | GAAAGCAGGUCAACUAUGA |
| ACSF3 | NM_174917 | 28372536 | CAAAUCAGGUCACGUAGAA |
| AFG3L2 | NM_006796 | 5802969 | GAAAUUCUCUUUUGACAGA |
| AGO1 | NM_012199 | 29171732 | GGAGUUACUUUCAUAGCAU |
| ALDH4A1 | NM_003748 | 25777733 | UCAAACACCUGUGGAAGCA |
| ALOX12B | NM_001139 | 119964724 | UGUCGGAGCUCACCUAUGA |
| ATP5F1 | NM_001688 | 85794838 | CGCCUGGACUAUCAUAUAU |
| ATXN2L | NM_007245 | 27262646 | CUUGUUCACUUCCGAAAUG |
| BCAP31 | NM_005745 | 49472837 | UGGAGUUGUUAGUGUCCUA |
| BCAS2 | NM_005872 | 49472833 | GAAACGAUAUGAGCUUCCA |
| BUB3 | NM_001007793 | 56550080 | GGACUGCGCCUUCUACGAU |
| CAD | NM_004341 | 47458828 | AUUAAGACUCCACGGGUAU |
| CANX | NM_001024649 | 66933004 | UGAUUGAUAUUGAGGAUGA |
| CAPRIN1 | NM_203364 | 61676202 | UAGUCAGCCUCACCAAGUA |
| CASP14 | NM_012114 | 6912285 | ACAGAUGCCUUGCACGUUU |
| CLPX | NM_006660 | 12597621 | CAACAAAUACCUCAGGAAA |
| CNOT8 | NM_004779 | 31542314 | GAAUAGCCAGGUUAUCUGU |
| COPG1 | NM_012133 | 109134348 | GAGAGCUGCUUGCGAAAUA |
| COPG2 | NM_138434 | 49574496 | GGAAGCAAGUCCUCGUGUA |
| COX5A | NM_004255 | 17017986 | GAUAUAGAUGCCUGGGAAU |
| CSDE1 | NM_007158 | 56117849 | AGGAUGGCAUUAUUGCUUA |
| CSE1L | NM_001316 | 29029558 | UGGUUUGUGUUGAGCGUUU |
| CSNK1A1 | NM_001892 | 68303571 | GCGAUGUACUUAAACUAUU |
| DAZAP1 | NM_018959 | 25470885 | CGGAGGUAGUCAUGAUCUA |
| DDX17 | NM_001098505 | 148613857 | CAAGGAUGGUGGCCGGAGA |
| DDX21 | NM_004728 | 50659094 | CAAACUAGAUCUCACCAAA |
| DHX30 | NM_014966 | 20336289 | GCGUGGAGGUAGAAGGCUA |
| DICER1 | NM_030621 | 29294648 | GAUCCUAUGUUCAAUCUAA |
| DLD | NM_000108 | 91199539 | GUACAAAGUUGGGAAAUUC |
| DNM2 | NM_004945 | 56549118 | AGUCCUACAUCAACACGAA |
| DOCK2 | NM_004946 | 31377467 | GAAUGAGUAUCGCUCCGUU |
| DYNC1H1 | NM_001376 | 94557306 | GGAUGAAUCUAAUGUGUUA |
| EIF3C | NM_003752 | 76443656 | ACGAAUGGAUGAAGAAUUU |
| EPRS | NM_004446 | 62241041 | GGAAACUGAUCAUGAGAUU |
| eRF1 | NM_006494 | 156104871 | GCGCUGGAGUGAAGACUGU |
| ETFB | NM_001985 | 62420878 | AAGUGGAGCGGGAGAUCGA |
| FAM120A | NM_014612 | 68299753 | GCGUAUGACUCUGAUUAUG |
| FAM133B | NM_152789 | 91206455 | CUGAAUCGACCAAGGCCUA |
| FTO | NM_001080432 | 122937262 | GAUGAUGUCUCUUUGAAAU |
| G3BP1 | NM_198395 | 38327551 | GUGCGAGAACAACGAAUAA |
| GALNT2 | NM_004481 | 9945385 | GGAAGUACGACAUGAUGAU |
| GANAB | NM_198334 | 38202256 | GGGCUAACAUGUUCAGCUA |
| GBAS | NM_001483 | 4503936 | GCCAAAGAUUCACGAAGAU |
| GCDH | NM_013976 | 50959158 | GGCCAAUCGCAACGAAGUU |
| GRSF1 | NM_001098477 | 149193318 | GGACUUCCUUAUAGUUGCA |
| GSDMA | NM_178171 | 150456462 | CCUAACAGAGCUAAGUGAA |
| H1F0 | NM_005318 | 85838503 | UCGAUUUGGGAUUUGCUAA |
| HADHA | NM_000182 | 105990523 | CGAAACAUGUGGCGGAAGA |
| HINT2 | NM_032593 | 49574527 | GAACCUGCCAACUGAUUAA |
| HIST1H1B | NM_005322 | 15718716 | GCUGCUAAGCGCAAAGCGA |
| HIST1H1D | NM_005320 | 20544161 | GAAGAAGGCAGGCGCAACU |
| HIST1H1E | NM_005321 | 20544164 | GAAGGCCAAAGCAGUUAAA |
| HIST1H1C | NM_005319 | 21071025 | UAACCAAGAAAGUGGCUAA |
| HNRNPA2B1 | NM_002137 | 156151373 | GCAAGACCUCAUUCAAUUG |
| HNRNPA3 | NM_194247 | 98961157 | UAUAAUGGAUUUGGAGGUG |
| HNRNPK | NM_002140 | 14165438 | GUCGGGAGCUUCGAUCAAA |
| HNRNPL | NM_001005335 | 52632384 | GGAGUGAAGCGGCCAUCUU |
| HNRNPR | NM_001102397 | 156151391 | CUCCAGAAGCACUGCGUAU |
| HSD17B10 | NM_001037811 | 91823617 | GUACAGGCCAUCAUCGAGA |
| IDH2 | NM_002168 | 28178831 | GACAUCCAGCUAAAGUAUU |
| IDH3B | NM_006899 | 28178820 | UUGAGACAAUGAUCAUAGA |
| IGF2BP1 | NM_006546 | 56237026 | CGGGAAAGUAGAAUUACAA |
| IGF2BP3 | NM_006547 | 30795211 | UCGAGGCGCUUUCAGGUAA |
| ILF2 | NM_004515 | 24234746 | GGACAUGGUCUGCUAUACA |
| IPO4 | NM_024658 | 62460636 | AUGGAGCACCUGCGGGAAU |
| KPNA2 | NM_002266 | 62388891 | GUAAAUUGGUCUGUUGAUG |
| LARS | NM_020117 | 108773809 | GGGAAAGCCUGACUCAAUU |
| LGALS9 | NM_002308 | 102469693 | GCAACACCCAGAUCGACAA |
| LOR | NM_000427 | 109255250 | GCUUAGAGCUCUCAUGAUG |
| LRPPRC | NM_133259 | 31621304 | UCGAAGAACUGGCCGCAUU |
| MBNL1 | NM_207294 | 46411167 | CCAUAAUAUCUGCCGAACA |
| MBOAT7 | NM_024298 | 23308571 | GGGAAACUGAGGUGCGGGU |
| MCCC1 | NM_020166 | 116805326 | GAAUGAGGAUUGUUAGAUC |
| METTL14 | NM_020961 | 34147341 | GCAUUGGUGCCGUGUUAAA |
| METTL3 | NM_019852 | 99077115 | CAGCACAGCUUCAGCAGUU |
| MRPL1 | NM_020236 | 143770801 | CGAGACAGAUAUAUGAGGU |
| MRPS31 | NM_005830 | 16950599 | GCACGGAACAGUCAGGUAC |
| MRPS9 | NM_182640 | 33188462 | GAAAGUUUACGUGGAAGAA |
| MTHFD1L | NM_015440 | 40018634 | GGAGAUGGCUUCGUGAACA |
| MTHFD2 | NM_006636 | 94721353 | GUUACAAUAUCUCAUCGAU |
| MUT | NM_000255 | 156105688 | UGUGAAAAGUGCUCGAAUU |
| NCAPG | NM_022346 | 50658080 | CAAUAUCCCUGGUUUCUUU |
| NCBP1 | NM_002486 | 110349725 | GUACACAACACUCCUCAUU |
| NCLN | NM_020170 | 24308184 | GGAAUGCAGUGCUGAACAC |
| NCOA5 | NM_020967 | 62953138 | ACUCUUAUUUUGACCGUUA |
| NUP155 | NM_004298 | 24430147 | CAUUUGGGAUGCAAGCUUA |
| NUP54 | NM_017426 | 26051236 | AUGAAGAGCAGUUGCGAGU |
| OAT | NM_000274 | 4557808 | GACCAUUUAUGCCGGGAUU |
| PABPC1 | NM_002568 | 56676313 | UGGAUGAGAUGAACGGAAA |
| PABPN1 | NM_004643 | 4758875 | UGACAAAUUUAGUGGCCAU |
| PC | NM_022172 | 106049291 | UCUCUGAGCGAGCGGACUU |
| PCCA | NM_000282 | 65506441 | GCAGUUGAAUGUCGGGUUU |
| PMPCB | NM_004279 | 94538353 | GAUUUAAGAAGUUGCGAUU |
| PRRC2A | NM_080686 | 149158689 | AGACUUACGCCUAGUGGUA |
| PRRC2C | NM_015172 | 115298681 | GGAUCUAACCAUACGCAAA |
| PSMD14 | NM_005805 | 5031980 | GUACUUAUGACCUCAAAUA |
| RBM15/RBM15B | NM_022768 | 22095383 | GGUGAUAGUUGGGCAUAUA |
| RBM4 | NM_002896 | 93277121 | GGGCUGAAAUUCCGAGCUG |
| RBMS1 | NM_002897 | 46249390 | ACAAGGAUACUACGUGAUU |
| RBMX | NM_002139 | 56699408 | GUGGAAGUCGAGACAGUUA |
| RFC3 | NM_181558 | 108773788 | CAAAUGCUAUUGUCAGUCA |
| RPL15 | NM_002948 | 15431292 | CGAAAACGCCCAGUUCCUA |
| RPL21 | NM_000982 | 78190465 | CCACAUAUAUGCGAAUCUA |
| RPL29 | NM_000992 | 17105395 | GCCAAGAAGCACAACAAAA |
| RPS17 | NM_001021 | 71772428 | AGGAGAUUAUUGAAGUAGA |
| SBNO1 | NM_018183 | 33620762 | GCAACUAGCUCGAGAGGAA |
| SCCPDH | NM_016002 | 55770835 | GCGUAGGACCAUAUCGGUU |
| SEPT2 | NM_004404 | 56550108 | GGUCGGUGAAUCAGGUCUA |
| SF3B2 | NM_006842 | 55749530 | AAGCGUAGGAACCGAAAGA |
| SLIRP | NM_031210 | 33667026 | UGACAAGGAGACUGGCUUU |
| SLTM | NM_001013843 | 62244003 | GGAACGCAUUCGUAUUGAA |
| SON | NM_032195 | 21040313 | CAAUGUCAGUGGAGUAUCA |
| SPEN | NM_015001 | 14790189 | CAUCGAGCAUUUCAAACGA |
| SRP72 | NM_006947 | 109638748 | AUAGAGAUCUCGUCCGAAA |
| SRRM2 | NM_016333 | 118572612 | GACAGCAAAUCUCGACUAU |
| SRSF6 | NM_006275 | 38158029 | GGAUACAGCAGUCGGAGAA |
| STAU2 | NM_014393 | 7657624 | GAAGGUAGGCAAUGAAGUU |
| TARS2 | NM_025150 | 39725684 | CGCCAGUUGUUCAAGGAUA |
| TCOF1 | NM_001008657 | 57164978 | CCUCAGAGCUUGGUCGGAA |
| TGM3 | NM_003245 | 39777600 | ACAAAGGCCUUGGCUCUAA |
| TNPO1 | NM_153188 | 133904027 | CAAUUGGUCGUCUUGGUUA |
| TOMM40 | NM_006114 | 5174722 | GCAAGAACAAGUUUCAGUG |
| TOP1 | NM_003286 | 19913404 | ACAUAAAGGUCCAGUAUUU |
| TRA2A | NM_013293 | 110227858 | UGGGCGAUCUCGAGGAUUU |
| TRIM56 | NM_030961 | 30794215 | GAUAAGAAGGGCUACAUCU |
| TUFM | NM_003321 | 34147629 | CAAGCCAGGUUCCAUCAAG |
| U2AF1 | NM_001025203 | 68800127 | GAGCGUCGUUCUCGGUCUA |
| UBAP2L | NM_014847 | 40254860 | CAACACAGCAGCACGUUAU |
| UPF1 | NM_002911 | 88853067 | CAAGAUAACAUCACUGUCA |
| UPF2 | NM_015542 | 18375674 | GGUCUAGAGAGUUGCGAAU |
| UPF3A | NM_023011 | 18375523 | AAAGAAACGUUUGCGGGAA |
| UPF3B | NM_023010 | 18375526 | CUAAUGAUACGAGUUUGUA |
| VAPA | NM_194434 | 94721251 | CAAGGAAACUAAUGGAAGA |
| VARS | NM_006295 | 94538367 | GAAGAGGGAGAAACGGGAU |
| VWA8 | NM_001009814 | 57863272 | UGGAAAAUUGUCCGAGUUA |
| YBX1 | NM_004559 | 109134359 | GUAAGGAACGGAUAUGGUU |
| YBX3 | NM_003651 | 21359983 | CAACGUCAGAAAUGGAUAU |
| YTHDC1 | NM_133370 | 94536804 | GGAAUUUCAUAACAUGGGA |
| YTHDC2 | NM_022828 | 38505212 | GGACUAGGAGGAGUAUUUA |
| YTHDF1 | NM_017798 | 31377749 | AAGGAACGGCAGAGUCGAA |
| YTHDF2 | NM_016258 | 116812574 | GCCAUGCCCUACUUAACUU |
| YTHDF3 | NM_152758 | 116235459 | ACAUACAUCGUUCCAUUAA |
| ZNF326 | NM_182975 | 156523256 | ACACUCCGCCUGCAGGAAU |
