# Supplementary material for: Identification of host proteins differentially associated with HIV-1 RNA splice variants
Source: eLife. 2021 Feb 25;10:e62470. doi: 10.7554/eLife.62470 (PMC7906601; doi:10.7554/eLife.62470)
Supplement: Supplementary file 5. — Related to Figures 4 and 5 and Figure 4—figure supplements 1–3. [file elife-62470-supp5.docx]

**Supplementary File 5:** Antibodies used for immunoblots and immunofluorescence. Related to Figures 4, 5 and Figure 4-figure supplements 1-3.

| **Protein Target** | **Supplier** | **Catalog Number** | **Conjugate** | **Dilution for Immuno-fluorescence** |
| --- | --- | --- | --- | --- |
| BUB3 | ThermoFisher Scientific | PA5-20388 | NA | 1:200 |
| CSDE1 | ThermoFisher Scientific | PA5-22394 | NA | 1:200 |
| DHX30 | ThermoFisher Scientific | PA5-41298 | NA | 1:200 |
| DLD | ThermoFisher Scientific | PA5-27367 | NA | 1:200 |
| DNM2 | ThermoFisher Scientific | PA1-661 | NA | 1:200 |
| DYNC1H1 | ThermoFisher Scientific | PA5-49451 | NA | 1:200 |
| FAM120A | ThermoFisher Scientific | PA5-54069 | NA | 1:200 |
| G3BP1 | Santa Cruz | SC-98561 | NA | 1:200 |
| GSDMA | ThermoFisher Scientific | PA5-24813 | NA | 1:200 |
| HNRNPR | ThermoFisher Scientific | PA5-55290 | NA | 1:200 |
| IGF2BP3 | ThermoFisher Scientific | PA5-51672 | NA | 1:200 |
| LRPPRC | ThermoFisher Scientific | PA5-22034 | NA | 1:200 |
| MBOAT7 | AbCam | ab105643 | NA | 1:200 |
| NCLN | ThermoFisher Scientific | PA5-34356 | NA | 1:200 |
| RBM4 | ThermoFisher Scientific | PA5-21755 | NA | 1:200 |
| RBMX | ThermoFisher Scientific | PA5-49468 | NA | 1:200 |
| RPL15 | ThermoFisher Scientific | PA5-48446 | NA | 1:200 |
| SRRM2 | ThermoFisher Scientific | PA5-59559 | NA | 1:200 |
| SRSF6 | ThermoFisher Scientific | PA5-56034 | NA | 1:200 |
| TRIM56 | ThermoFisher Scientific | MA5-27066 | NA | 1:200 |
| Gag/p24 | NIH AIDS Reagent Program | 183-H12-5C | NA | 1:200 |
| Goat anti-Rabbit secondary antibody | ThermoFisher Scientific | A11008 | Conjugate Alexa Fluor 488 | 1:500 |
| Goat anti-Mouse secondary antibody | ThermoFisher Scientific | A21235 | Conjugate Alexa Fluor 647 | 1:500 |
| Goat anti-Rabbit secondary antibody | LiCor Biosciences | 926-32211 | LiCor IRDye800 |  |
| Goat anti-Mouse secondary antibody | LiCor Biosciences | 926-68020 | LiCor 680LT |  |
