# Supplementary material for: Identification of host proteins differentially associated with HIV-1 RNA splice variants
Source: eLife. 2021 Feb 25;10:e62470. doi: 10.7554/eLife.62470 (PMC7906601; doi:10.7554/eLife.62470)
Supplement: Supplementary file 7. — Related to Figures 4 and 5, Figure 4—figure supplements 1–3. [file elife-62470-supp7.docx]

**Supplementary File 7:** Stellaris-designed FISH probes specific to US HIV RNA. Related to Figures 4, 5, Figure 4-figure supplement 1-3.

| **Sequence** | **Three Modification** |
| --- | --- |
| aactgcgaatcgttctagct | T(CAL Fluor Red 590) |
| atgtctctaaaaggccagga | T(CAL Fluor Red 590) |
| tgaagggatggttgtagctg | T(CAL Fluor Red 590) |
| tcttatctaaggcttccttg | T(CAL Fluor Red 590) |
| tactacttttacccatgcat | T(CAL Fluor Red 590) |
| acatgggtattacttctggg | T(CAL Fluor Red 590) |
| catgcactggatgcaatcta | T(CAL Fluor Red 590) |
| atcctatttgttcctgaagg | T(CAL Fluor Red 590) |
| gggataggtggattatgtgt | T(CAL Fluor Red 590) |
| tggtagggctatacattctt | T(CAL Fluor Red 590) |
| ctcggctcttagagttttat | T(CAL Fluor Red 590) |
| ggtttctgtcatccaatttt | T(CAL Fluor Red 590) |
| caatctgggttcgcattttg | T(CAL Fluor Red 590) |
| ttcagccaaaactcttgctt | T(CAL Fluor Red 590) |
| agtctttctttggttcctaa | T(CAL Fluor Red 590) |
| caaacctgaagctctcttct | T(CAL Fluor Red 590) |
| gagtgatctgagggaagcta | T(CAL Fluor Red 590) |
| tccgcagatttctatgagta | T(CAL Fluor Red 590) |
| gtcctactaatactgtacct | T(CAL Fluor Red 590) |
| ccattgtttaacttttgggc | T(CAL Fluor Red 590) |
| cccagaaatcttgagttctc | T(CAL Fluor Red 590) |
| gcagtatacttcctgaagtc | T(CAL Fluor Red 590) |
| tggaatattgctggtgatcc | T(CAL Fluor Red 590) |
| atgttttttgtctggtgtgg | T(CAL Fluor Red 590) |
| gatggagttcataacccatc | T(CAL Fluor Red 590) |
| ccctgcataaatctgacttg | T(CAL Fluor Red 590) |
| gtactacttctgttagtgct | T(CAL Fluor Red 590) |
| tagaatctccctgttttctg | T(CAL Fluor Red 590) |
| taatacactccatgtaccgg | T(CAL Fluor Red 590) |
| tgcttctgtatttctgctat | T(CAL Fluor Red 590) |
| cattcttgcatactttcctg | T(CAL Fluor Red 590) |
| tctttccccatattactatg | T(CAL Fluor Red 590) |
| catgtttccttttgtatggg | T(CAL Fluor Red 590) |
| ctaagggaggggtattgaca | T(CAL Fluor Red 590) |
| cgttagggggacaacttttt | T(CAL Fluor Red 590) |
| tgcttgtaactcagtcttct | T(CAL Fluor Red 590) |
| ttacttctaatcccgaatcc | T(CAL Fluor Red 590) |
| gtgcttgaatgattcccaat | T(CAL Fluor Red 590) |
| ttgttcatttcctccaattc | T(CAL Fluor Red 590) |
| atagtactttcctgattcca | T(CAL Fluor Red 590) |
| gccttatctattccatctaa | T(CAL Fluor Red 590) |
| aaatcactagccattgctct | T(CAL Fluor Red 590) |
| tggctactatttcttttgct | T(CAL Fluor Red 590) |
| cccttttagctgacatttat | T(CAL Fluor Red 590) |
| tacatgaactgccaccaaga | T(CAL Fluor Red 590) |
| tggtgaaattgctgccattg | T(CAL Fluor Red 590) |
| actttggggattgtagggaa | T(CAL Fluor Red 590) |
| tctgctgtccctgtaataaa | T(CAL Fluor Red 590) |
